# Essential oil massage and incident chronic kidney disease–associated pruritus in maintenance hemodialysis patients without baseline pruritus: a prospective non-concurrent before-and-after study

**DOI:** 10.3389/fmed.2026.1848586

**Published:** 2026-09-15

**Authors:** Xian Shen, Ting Shen, Jing Luo, Peng Fu, Mengru Liu, Congcong Cao, Hao Wu, Jing Cui

**Affiliations:** 1 Shidong Hospital, University of Shanghai for Science and Technology, Shanghai, China; 2 School of Nursing, Bengbu Medical College, Bengbu, China

**Keywords:** chronic kidney disease-associated pruritus, essential oil massage, hemodialysis, non-pharmacological intervention, symptom management

## Abstract

**Objective:**

To compare the incidence of chronic kidney disease-associated pruritus (CKD-aP) between two consecutive care phases among maintenance hemodialysis patients without pruritus at baseline, following the addition of essential oil massage to standardized hemodialysis care and basic skin care.

**Methods:**

This single-center, prospective, non-concurrent before-and-after controlled study enrolled 87 maintenance hemodialysis patients from January to December 2024. Patients in the control phase (*n* = 43) received routine dialysis care and basic skin care, whereas those in the intervention phase (*n* = 44) received additional essential oil massage. The primary outcome was incident pruritus. Topical ointment use was evaluated as an exploratory secondary outcome. Exploratory analyses among patients who developed pruritus during follow-up included pruritus severity assessed using the visual analog scale (VAS) and duration of ointment use. Skin-related adverse reactions were also recorded in the intervention group.

**Results:**

No baseline characteristic differed statistically between the two phases; however, SMDs indicated mild imbalance in several variables. During follow-up, the incidence of pruritus was significantly lower in the intervention group than in the control group (31.8% vs. 65.1%, *P* = 0.002). The rate of topical ointment use was also significantly lower in the intervention group (13.6% vs. 48.8%, *P* < 0.001). Among patients who developed pruritus, the intervention group showed lower VAS scores and shorter duration of ointment use than the control group (both *P* < 0.01). Two patients (4.5%) in the intervention group developed mild local adverse reactions, all of which resolved after temporary discontinuation or symptomatic management.

**Conclusion:**

The intervention phase showed a lower observed incidence of new-onset CKD-aP and less topical ointment use among maintenance hemodialysis patients without pruritus at baseline. However, because intervention exposure was completely aligned with calendar period, this study cannot distinguish an intervention effect from a seasonal effect, and the observed difference should not be interpreted as evidence of preventive benefit. Findings regarding pruritus severity among patients who developed pruritus during follow-up were exploratory.

## 1 Introduction

Chronic kidney disease-associated pruritus (CKD-aP) is a common and distressing symptom among patients undergoing maintenance hemodialysis. In DOPPS phases 4–6, 37% of patients receiving hemodialysis reported being at least moderately bothered by pruritus, with substantial variation across countries (1). CKD-aP not only impairs patient comfort but is also associated with sleep disturbances, depressive symptoms, reduced health-related quality of life, prolonged post-dialysis recovery, poor dialysis adherence, and adverse clinical outcomes. It is therefore regarded as a major clinical concern in symptom management for dialysis patients (2).

The pathogenesis of CKD-aP is not fully understood. A narrative review by Rigatto et al. summarized several proposed and potentially interacting mechanisms of CKD-aP, including uremic toxin accumulation, immune and inflammatory dysregulation, peripheral and central neural dysfunction, opioid-receptor imbalance, xerosis, and impaired skin-barrier function (3). Given its multifactorial etiology, clinical management generally requires a comprehensive approach. Current expert opinions and reviews recommend that, after identifying and excluding other causes of pruritus, initial management should focus on optimizing dialysis adequacy and metabolic parameters including calcium, phosphorus, and parathyroid hormone, combined with intensive skin moisturization and basic non-pharmacological care. For patients with moderate-to-severe symptoms, gabapentin, pregabalin, or *κ*-opioid receptor agonists may be considered (4). By contrast, the level of evidence and strength of recommendations for antihistamines, topical calcineurin inhibitors, and other similar interventions remain relatively limited.

Against this background, the value of adjunctive non-pharmacological interventions has gained increasing attention. Basic skin care, particularly regular emollient use to improve skin hydration, is considered a cornerstone of CKD-aP management. Meanwhile, aromatherapy massage—an adjunctive intervention combining tactile stimulation, topical care, and sensory relaxation—has attracted growing interest in the management of chronic symptoms (5). Previous small-scale studies have suggested that certain essential oils or topical preparations containing botanical active ingredients may help relieve pruritus, improve xerosis, and enhance sleep quality. However, existing evidence is largely limited to small, short-term studies with considerable methodological heterogeneity, and the adjunctive effectiveness of such interventions for CKD-aP in maintenance hemodialysis patients requires further validation in more rigorously designed studies (3, 6).

Accordingly, this study adopted a single-center, prospective, non-concurrent before-and-after design to compare the incidence of CKD-aP and topical symptomatic medication use between two consecutive care phases among maintenance hemodialysis patients without pruritus at baseline. The primary aim was to explore whether the addition of essential oil massage to standardized hemodialysis care and basic skin care was associated with a lower incidence of new-onset CKD-aP. Topical ointment use was evaluated as an exploratory secondary outcome, and pruritus severity and duration of ointment use among patients who developed pruritus during follow-up were evaluated in exploratory analyses.

## 2 Methods

### 2.1 Study participants

Patients who received maintenance hemodialysis at the Hemodialysis Center of Shidong Hospital from January 2024 to December 2024 were enrolled as study participants. According to the timing of the intervention, the study was divided into two consecutive phases: the control phase from January to June 2024, and the intervention phase from July to December 2024. During the control phase, patients received routine dialysis care and basic skin care, while during the intervention phase, essential oil massage was added on top of the same basic care.

**Inclusion criteria:** (1) age ≥18 years; (2) receiving regular maintenance dialysis three times per week for ≥3 months, with a fixed dialysis regimen consisting of 4 sessions of hemodiafiltration (HDF) combined with 9 sessions of low-flux hemodialysis per month; (3) fully conscious and able to cooperate with study observations and data collection; (4) VAS score of 0 (no pruritus) at enrollment; (5) signed informed consent and voluntarily participated in the study.

**Exclusion criteri**a: (1) presence of a dermatological disease or a systemic condition other than chronic kidney disease that could independently cause pruritus; (2) allergy to essential oils, carrier oils, or topical ointment ingredients; (3) severe dysfunction of heart, liver, lungs, or other vital organs, or malignancy; (4) cognitive impairment, psychiatric disorders, or poor compliance preventing completion of observations.

No random, systematic, or referral sampling was used. All 87 enrolled participants were distinct individuals, and no participant contributed observations to both study phases. Each participant was enrolled only once according to the calendar phase in which eligibility was first assessed and consent was obtained; participants enrolled during the control phase were not re-enrolled during the intervention phase. At the start of the intervention phase, the January–June enrollment log was cross-referenced with the July dialysis roster to identify and exclude patients who had already been enrolled during the control phase. Continued receipt of maintenance hemodialysis alone did not result in automatic enrollment into the intervention phase.

#### 2.1.1 Ethics statement

The studies involving human participants were reviewed and approved by the Medical Ethics Committee of Shidong Hospital, University of Shanghai for Science and Technology, Shanghai, China (Approval No. 2024-041-01). Written informed consent was obtained from all participants prior to enrollment.

### 2.2 Study design

This was a single-center, prospective, non-randomized, non-concurrent before-and-after quasi-experimental nursing intervention study. Prospective clinical trial registration was not performed before enrollment, and retrospective trial registration was not pursued. Before study commencement, the complete study protocol, eligibility criteria, predefined outcomes, and statistical analysis plan were prospectively reviewed and formally approved by the institutional ethics committee (Ethics No. 2024-041-01), and the full protocol was archived in the institutional research files. This study was reported with reference to the Transparent Reporting of Evaluations with Nonrandomized Designs (TREND) statement. A completed TREND checklist with corresponding page numbers is provided as Supplementary Material. All eligible patients were assigned to study phases according to the enrollment period. Patients in the control phase received routine dialysis care and basic skin care, whereas patients in the intervention phase received essential oil massage in addition to the same basic care. Each participant was followed for a fixed period of 4 weeks from the date of enrollment, regardless of the calendar date of enrollment. Participants remained under the care protocol assigned at enrollment throughout the 4-week follow-up; therefore, the scheduled time at risk was equal between the two study phases.

### 2.3 Intervention methods

All patients in both phases received standardized dialysis treatment and uniform basic skin care. Control phase patients received standardized dialysis treatment combined with basic skin care; intervention phase patients received essential oil massage in addition. To reduce bias from time trends and co-interventions in the non-concurrent before-and-after design, both phases were managed by the same nursing team, using consistent dialysis prescriptions, nursing education content, follow-up frequency, and outcome evaluation procedures. Any adjustments to dialysis parameters or additional treatments due to patient condition were evaluated by the responsible physician and fully recorded.

The intervention was conceptually based on two complementary pathways: the oil-based preparation was intended to support local skin hydration and barrier integrity, while gentle massage was intended to provide tactile input that might modulate itch-related sensory processing. These pathways were theoretical and were not directly measured in this study.

#### 2.3.1 Common background treatments

##### 2.3.1.1 Standardized dialysis treatment

Patients in both phases underwent hemodialysis using the same platform. Dialysis equipment, dialysate supply system, and main dialysis parameters were consistent throughout the study. The standard dialysis prescription was three sessions per week, 4 h each, dialysate flow 500 mL/min, and blood flow 200–250 mL/min. Ultrafiltration volume was individualized according to dry weight, volume status, and clinical assessment, but no systematic adjustment based on study group was performed. Blood pressure, pulse, dialysis adequacy, and dialysis-related adverse events were routinely monitored.

##### 2.3.1.2 Uniform basic skin care

All patients received standardized basic skin care guidance from trained responsible nurses, with one-on-one instruction at baseline and reinforcement during follow-up. Care included avoiding hot water or frequent use of alkaline cleansers, avoiding excessive scratching or mechanical irritation, wearing loose, soft, breathable clothing, maintaining suitable room temperature and humidity, and timely moisturizing after cleansing.

A non-medicated, fragrance-free moisturizer was provided, with instructions to apply once in the morning and evening and preferentially after bathing or when skin dryness was evident. For patients with significant dryness, additional applications were permitted under nurse guidance. To ensure consistency, the type of moisturizer, usage method, education content, and records were kept uniform across both phases.

##### 2.3.1.3 Compliance and co-intervention management

During the study, responsible nurses recorded patient adherence to skin care, including frequency of moisturizer use, bathing habits, skin abrasions or damage, and any new discomfort, via face-to-face dialysis visits or phone follow-ups. Patients were discouraged from using other topical antipruritic agents, herbal washes, steroid creams, or new sedatives outside the study protocol. Any additional treatments due to symptom changes were recorded, including drug name, dosage, start time, and duration, and considered as co-intervention factors in statistical analysis.

#### 2.3.2 Control group

Control phase patients received routine follow-up management on top of standardized dialysis treatment and basic skin care, without essential oil massage. Responsible nurses collected pruritus symptoms, skin condition, and related treatment data, and recorded adherence, symptom changes, and adverse events. Symptom monitoring and outcome evaluation procedures were identical to the intervention phase. If patients developed pruritus or worsening symptoms, symptomatic treatment was provided per protocol, and patients continued follow-up.

#### 2.3.3 Intervention group

Intervention phase patients received essential oil massage in addition to standardized dialysis treatment and basic skin care. Although caregiver availability was not specified as a formal inclusion criterion, implementation of the intervention required the availability of a designated family caregiver who was able and willing to perform the prescribed massage.

##### 2.3.3.1 Essential oil composition and preparation

The essential-oil preparation consisted of lavender (Lavandula angustifolia), German chamomile (Matricaria chamomilla), and tea tree (Melaleuca alternifolia) essential oils mixed at a ratio of 1:1:1. The essential-oil mixture was diluted in sweet almond oil to a final total essential-oil concentration of 5% (v/v). Accordingly, the final preparation contained approximately 1.67% lavender oil, 1.67% German chamomile oil, and 1.66% tea tree oil, with sweet almond oil constituting the remaining 95%. For example, 100 mL of the final preparation contained 1.67 mL of lavender oil, 1.67 mL of German chamomile oil, 1.66 mL of tea tree oil, and 95 mL of sweet almond oil. The preparation was dispensed into amber glass bottles and stored in a cool, dark environment to minimize oxidation. Oils from the same batch were used whenever possible to maintain consistency. Tea tree oil was included for its potential antimicrobial and anti-inflammatory properties, although it is not an established treatment for CKD-aP and experimental evidence for its anti-inflammatory effects on human skin remains limited; the preparation was therefore conceptualized as a composite aromatherapy intervention rather than a tea-tree-oil-specific treatment. The total essential-oil concentration of 5% (v/v) was selected to balance potential activity with safety. Each individual oil was present at approximately 1.67%, below the concentrations commonly associated with increased irritation or sensitization risk in leave-on topical applications. In addition, application was restricted to approximately 9% of total body surface area, a 24-hour tolerance test was required before initiation, and adverse skin reactions were actively monitored with predefined discontinuation criteria. These safeguards were intended to mitigate the recognized contact-sensitization risk of oxidized tea tree oil in a leave-on, repeated-use product, consistent with general safety principles for aromatic topical preparations.

##### 2.3.3.2 Intervention procedure

Essential oil massage was performed by designated family caregivers who had received standardized training from the research nurses. Before each session, the hands and treatment area were cleaned, and damaged or infected skin, recent puncture sites, and arteriovenous fistula sites were avoided. For participants without pruritus, the non-fistula upper extremity, including the upper arm, forearm, and dorsum of the hand, was used as the predefined application area because the extremities are commonly affected by CKD-aP. Essential oil massage was initiated at enrollment and continued throughout the assigned 4-week intervention period. Approximately 3–5 mL in total of the diluted essential-oil preparation was applied during each session and distributed evenly over the predefined area. The treated area was limited to one upper extremity, corresponding to a maximum of approximately 9% of the total body surface area. The area was massaged gently using circular and linear movements for 15–20 min, once daily, at least 5 days per week. If incident pruritus occurred during follow-up, the assigned massage protocol was maintained to preserve intervention continuity, but the application site was adjusted to the predominant symptomatic region. The total amount of preparation remained 3–5 mL per session and the treated area did not exceed approximately 9% of the total body surface area. If pruritus involved multiple or widespread regions, massage was limited to the most symptomatic region. The intervention was discontinued if an adverse skin reaction or another prespecified discontinuation criterion occurred.

##### 2.3.3.3 Initial tolerance assessment

A small patch of diluted essential oil was applied to the inner forearm or behind the ear and observed for 24 h. No erythema, papules, burning, stinging, or worsened pruritus indicated tolerance; any significant reaction led to discontinuation and classification as intolerant. This 24-hour open application was an irritation/tolerance screen rather than a sensitization test and could not exclude delayed hypersensitivity.

##### 2.3.3.4 Consistency control

Standardized operation protocols including dosage, site, duration, frequency, and pause criteria were provided, with written cards given to patients. Adherence was assessed during regular nurse follow-up based on patient- or caregiver-reported intervention frequency, duration, massage sites, and missed or interrupted sessions. Retraining was provided when deviations from the standardized procedure were identified. However, session-level completion data were not systematically recorded, and no electronic or sensor-based objective monitoring method was used.

#### 2.3.4 Symptomatic treatment

Both groups received standardized symptomatic treatment for safety and ethics. Patients who developed pruritus could receive topical ointments when clinically indicated by the attending physician, with detailed records of use. The topical ointments were prescribed by attending physicians according to routine clinical practice and individual patient needs rather than being protocol-specified. These included emollient-based moisturizers, weak-to-moderate potency topical corticosteroids (e.g., hydrocortisone butyrate), and occasionally topical antihistamines or calcineurin inhibitors, depending on the severity and characteristics of the skin lesions. Because the choice, class, and potency of these preparations were not standardized, ointment use was treated as an exploratory outcome. At enrolment, participants' medication use was assessed by review of medical records and medication reconciliation with the patient. No participant was receiving systemic antipruritic therapy, including antihistamines, gabapentinoids, or *κ*-opioid receptor agonists, at baseline. These medications were initiated only after the occurrence of pruritus events, when clinically indicated, and were therefore not present at enrolment.

#### 2.3.5 Adverse event monitoring and management

Skin reactions temporally associated with essential-oil exposure were actively evaluated only during the intervention phase. These assessments focused on local erythema, papules, burning, stinging, worsened dryness, contact dermatitis, or other dialysis-related discomforts. Mild reactions after essential oil use could pause intervention and be reassessed; symptoms could resume with reduced dosage or shorter duration. Severe allergic reactions, persistent inflammation, or intolerable symptoms led to termination of essential oil intervention, but follow-up continued. All events were assessed for severity and relation to intervention and documented.

### 2.4 Outcome measure

#### 2.4.1 Primary outcome

Pruritus occurrence: Incident pruritus was defined as any new patient-reported itching during the preceding 7 days at a weekly assessment among participants who were free of pruritus at baseline, provided that the symptom could not be explained by a primary dermatological disease or another non-CKD-related cause. The number and proportion of participants who developed incident pruritus during the 4-week follow-up period were recorded. This was a study-specific binary definition rather than a validated CKD-aP assessment instrument.

#### 2.4.2 Secondary outcome

1. Exploratory pruritus severity: VAS scores were assessed only among patients who developed incident pruritus during follow-up.
2. Exploratory ointment usage rate: number and proportion of patients using topical ointments for pruritus.
3. Exploratory duration of ointment use: number of days each patient used topical ointments.
4. Skin-related adverse reactions: occurrence of local erythema, stinging, burning, or rash in the intervention group after essential oil use.

### 2.5 Data collection

Fixed responsible nurses collected demographic and clinical data including gender, age, dialysis duration, and primary disease. Laboratory parameters, including serum calcium, serum phosphorus, and serum albumin, as well as history of pruritus prior to enrollment, were obtained through retrospective review of electronic medical records at baseline. Kt/V was not included in the prespecified data collection protocol and was therefore unavailable for analysis. Weekly records included occurrence of pruritus, ointment use, and local adverse reactions. However, the exact week of first pruritus onset was not retained in an analyzable form, precluding reconstruction of week-specific cumulative incidence curves. VAS scores and ointment use were recorded for patients who developed pruritus. Information on intervention implementation was collected through patient or caregiver self-report and regular nurse follow-up. However, the numbers of planned and completed sessions were not recorded in a form that allowed calculation of the overall adherence rate or percentage of completed sessions. Accordingly, the analysis estimates the effect of assignment to the intervention rather than the effect of the intervention as delivered, and the possibility that some participants completed none of the assigned sessions cannot be excluded. Because study phase was determined by calendar period, the nursing assessors were aware of phase assignment and the study hypothesis. No independent or blinded outcome assessor was used.

### 2.6 Sample size and participant enrolment

No formal *a priori* sample size calculation was performed. At the time of study initiation, no independent pre-study estimate of the 4-week incidence of new-onset CKD-aP in maintenance hemodialysis patients without pruritus at baseline was available to support a reliable effect-size assumption. Therefore, no target sample size based on an expected between-group difference was prespecified.

Participants were enrolled according to the predefined eligibility criteria and consent procedures during the two prespecified study periods. The control phase was conducted from January to June 2024 and the intervention phase from July to December 2024. During these periods, 93 patients were screened and 87 eligible participants were enrolled, including 43 in the control phase and 44 in the intervention phase. The resulting sample size was therefore determined by the number of eligible and consenting participants enrolled during the predefined study periods rather than by a formal power calculation.

### 2.7 Statistical analysis

SPSS 22.0 was used. Normally distributed continuous variables were expressed as mean ± SD and compared using independent-sample *t*-tests; non-normally distributed variables as median (IQR) and compared using Mann–Whitney *U*-tests. VAS scores and duration of ointment use were analyzed only among patients who developed pruritus during the observation period. Patients without pruritus were excluded from these conditional analyses. Categorical variables were expressed as frequency and percentage, compared using *χ*^2^ or Fisher's exact test. Relative risks (RRs) and their 95% confidence intervals (CIs) were calculated using the Katz logarithmic method. Risk differences (RDs) and their 95% CIs were calculated using the Wald method. The number needed to treat (NNT) was calculated as the reciprocal of the absolute risk reduction. *P* < 0.05 was considered statistically significant. Standardized mean differences (SMDs) were calculated to quantify baseline imbalance between the two groups; an SMD greater than 0.1 was considered to indicate potential imbalance. Additionally, a multivariable logistic regression model was fitted as a sensitivity analysis to assess whether the between-phase difference in incident pruritus persisted after adjustment for baseline covariates. The model included incident pruritus as the dependent variable and group assignment, history of pruritus, serum phosphorus, and primary disease as independent variables. Primary disease was treated as a nominal categorical variable and entered using indicator (dummy) coding, with chronic glomerulonephritis specified as the reference category. Adjusted odds ratios (ORs) with 95% confidence intervals (CIs) were reported.

## 3 Results

### 3.1 Subject Enrollment

From January 2024 to December 2024, 93 patients undergoing maintenance hemodialysis at the Hemodialysis Center of Shidong Hospital were formally logged as screened for eligibility. This count does not necessarily include patients who were not enrolled during the intervention phase because an available family caregiver could not be identified. Six patients were excluded before enrollment because they did not meet the eligibility criteria; reason-level counts were not retained in the source records and could not be retrospectively reconstructed. The calendar-phase distribution of these six exclusions was likewise not retained and could not be reconstructed retrospectively. Caregiver-related non-enrollment during the intervention phase was not systematically tracked and may have constituted a separate category additional to the six documented exclusions. Eighty-seven participants were ultimately enrolled, including 43 in the control phase and 44 in the intervention phase. All 87 enrolled participants completed the prespecified 4-week follow-up, with no withdrawals or losses to follow-up. The final analysis included 43 participants in the control phase and 44 in the intervention phase. Participant flow is shown in Figure 1.

**Figure 1:**
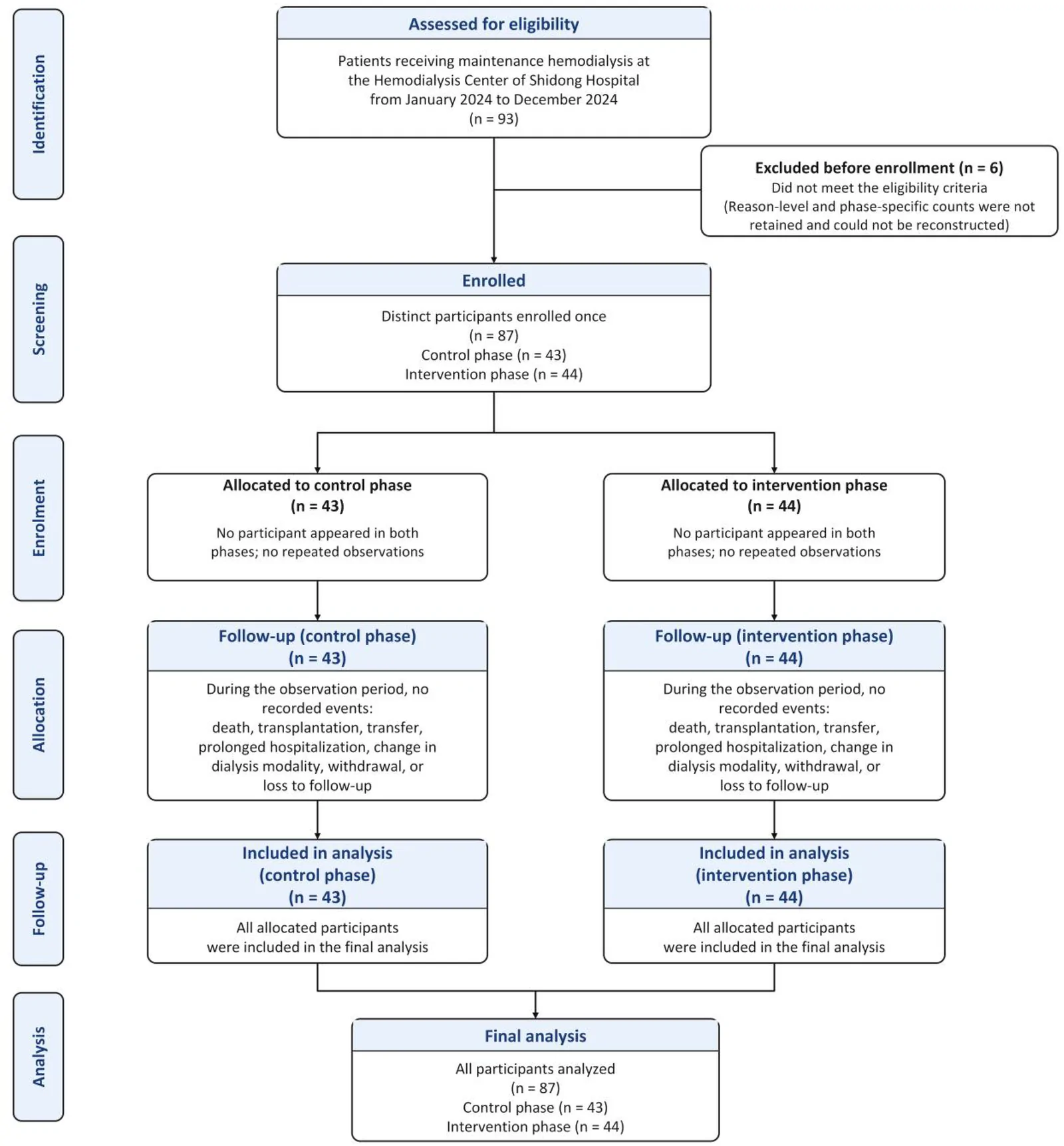
TREND-style participant flow through the study phases. The flow diagram summarizes patients recorded in the formal screening log; untracked caregiver-related non-enrollment during the intervention phase may not be represented.

### 3.2 Comparison of baseline characteristics between the two groups

Comparisons of baseline characteristics, including sex, age, dialysis vintage, primary disease distribution, serum calcium, serum phosphorus, serum albumin, history of pruritus, URR, intact parathyroid hormone (iPTH), C-reactive protein, and dialyzer membrane type, showed no statistically significant differences between the two groups (all *P* > 0.05). However, standardized mean difference (SMD) analysis revealed mild baseline imbalance in several variables (SMD > 0.1), including sex (SMD = 0.207), URR (SMD = 0.181), dialysis vintage (SMD = 0.157), primary disease distribution (SMD = 0.139), and serum calcium (SMD = 0.117). Given the non-randomized, non-concurrent before-and-after design, these variables may represent potential confounders and should be considered when interpreting the between-phase differences. See Table 1.

**Table 1:** Comparison of baseline characteristics between the two groups of patients.

Characteristic	Intervention Group	Control Group	Statistic *t*/Z/*χ*^2^	*P*-value	*SMD*
Age (years)	56.84 ± 7.29	56.35 ± 6.42	0.334	0.739	0.072
Gender [*n* (%)]			0.925	0.336	0.207
Male	25 (56.8)	20 (46.5)			
Female	19 (43.2)	23 (53.5)			
Dialysis vintage (years)	6.00 (6.00,7.00)	6.00 (5.00,7.00)	−0.422	0.673	0.157
Primary disease [*n* (%)]			0.421	0.936	0.139
Chronic glomerulonephritis	18 (40.9)	20 (46.5)			
Diabetic nephropathy	10 (22.7)	10 (23.3)			
Hypertensive nephropathy	9 (20.5)	7 (16.3)			
Others	7 (15.9)	6 (14.0)			
Serum calcium (mmol/L)	2.23 (2.15,2.33)	2.28 (2.13,2.40)	−0.641	0.521	0.117
Serum phosphorus (mmol/L)	1.69 ± 0.50	1.67 ± 0.50	0.254	0.800	0.054
Serum albumin (g/L)	36.47 ± 4.26	36.57 ± 3.80	−0.111	0.912	0.024
URR	66.17 ± 7.80	67.73 ± 9.37	0.845	0.401	0.181
iPTH	533.12 ± 280.30	506.32 ± 312.38	−0.421	0.675	0.090
C-reactive protein	6.98 ± 4.69	7.32 ± 4.82	0.315	0.754	0.067
Dialyzer membrane type [*n* (%)]			0.081	0.996	0.019
PS	26 (59.1)	25 (58.1)			
PES	12 (27.3)	12 (27.9)			
CTA	6 (13.6)	6 (14.0)			
History of pruritus [*n* (%)]			0.110	0.740	0.071
Yes	23 (52.3)	24 (55.8)			
No	21 (47.7)	19(44.2)			

Independent-samples *t*-tests were used for age, serum phosphorus, serum albumin, URR, iPTH, and C-reactive protein; Mann–Whitney *U*-tests (Z statistic) were used for dialysis vintage and serum calcium; Pearson's χ^2^-tests were used for sex, primary disease, dialyzer membrane type, and history of pruritus.

### 3.3 Comparison of incident pruritus and topical ointment use between the two study phases

During the observation period, incident pruritus occurred in 28 patients during the control phase and 14 patients during the intervention phase, corresponding to incidence rates of 65.1% and 31.8%, respectively. The incidence of new-onset pruritus was lower during the intervention phase than during the control phase (*χ*^2^ = 9.656, *P* = 0.002), with an RR of 0.49 (95% CI: 0.30–0.79), an RD of −33.30% (95% CI: −53.11% to −13.49%), and an NNT of 3.00 (Table 2; Figure 2).

**Table 2:** Comparison of incident pruritus and topical ointment use between the two study phases.

Item	Intervention Group	Control Group	Statistic *χ²*	*P*-value	RR (95% CI)	RD (95% CI)	NNT
Incident pruritus (*n* %)			9.656	0.002	0.49 (0.30–0.79)	−33.30% (−53.11% to −13.49%)	3.00
Yes	14 (31.8)	28 (65.1)					
No	30 (68.2)	15 (34.9)					
Topical ointment use (*n* %)			12.590	<0.001	0.28 (0.12–0.62)	−35.20% (−53.26% to −17.14%)	2.84
Yes	6 (13.6)	21 (48.8)					
No	38 (86.4)	22 (51.2)					

RR, relative risk; CI, confidence interval; RD, risk difference; NNT, number needed to treat. RDs were calculated as the intervention-phase risk minus the control-phase risk; therefore, negative values indicate lower risk during the intervention phase.

**Figure 2:**
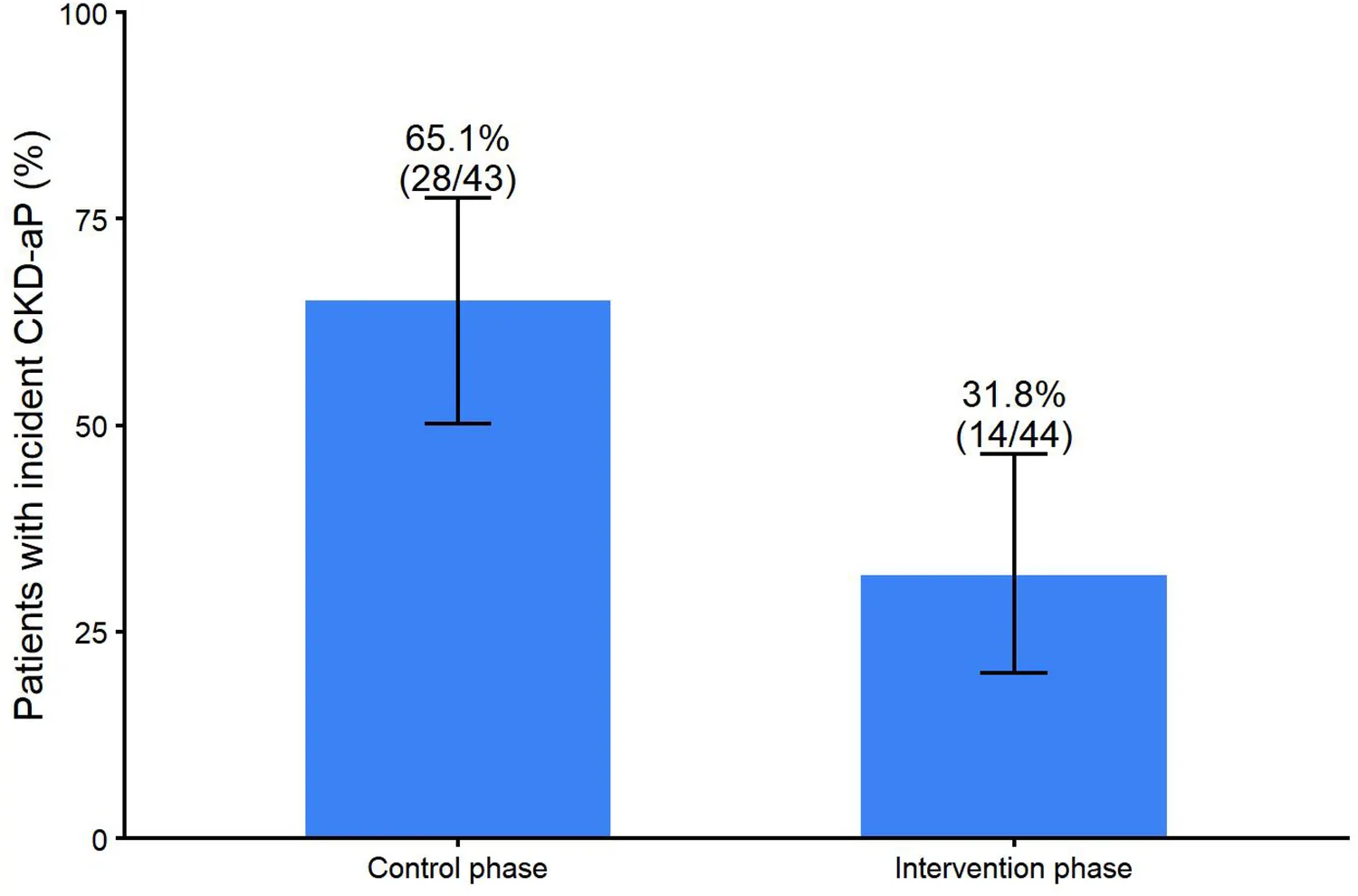
Incidence of CKD-aP during the 4-week follow-up in the control and intervention phases. Values above the bars represent the observed proportions and numbers of participants with incident CKD-aP.

In a multivariable logistic regression sensitivity analysis adjusted for history of pruritus, serum phosphorus, and primary disease, the intervention phase remained significantly associated with lower odds of incident pruritus (adjusted OR = 0.217, 95% CI: 0.084–0.559; *P* = 0.002). Primary disease was treated as a nominal categorical variable using dummy coding, with chronic glomerulonephritis as the reference category. The overall effect of primary disease was not statistically significant (Wald *χ*^2^ = 4.132, df = 3, *P* = 0.248). Among the category-specific contrasts, diabetic nephropathy was associated with higher odds of incident pruritus compared with chronic glomerulonephritis (adjusted OR = 4.042, 95% CI: 1.031–15.849; *P* = 0.045), whereas hypertensive nephropathy and other primary diseases were not statistically significant. History of pruritus and serum phosphorus were also not statistically significant. The association between study phase and incident pruritus therefore remained statistically significant after adjustment for these measured baseline covariates (Table 3).

**Table 3:** Multivariable logistic regression sensitivity analysis for incident pruritus.

variable	B	S.E.	*P*	Exp (B)	95%CIforEXP (B)	
					Lower	Upper
Serum phosphorus (mmol/L)	−0.124	0.495	0.802	0.883	0.335	2.329
History of pruritus	0.474	0.475	0.318	1.607	0.633	4.079
Group (1 = Intervention Group; 0 = Control Group)	−1.529	0.483	0.002	0.217	0.084	0.559
Primary disease, overall	*Overall Wald test: χ²* *=* *4.132, df* *=* *3, P* *=* *0.248*					
Chronic glomerulonephritis	Reference					
Hypertensive nephropathy	0.174	0.604	0.773	1.191	0.364	3.893
Diabetic nephropathy	1.397	0.697	0.045	4.042	1.031	15.849
Other	0.255	0.701	0.716	1.290	0.327	5.096

Primary disease was treated as a nominal categorical variable using indicator coding (dummy-variable coding), with chronic glomerulonephritis as the reference category. The overall Wald test for primary disease was χ^2^ = 4.132, df = 3, *P* = 0.248. OR, odds ratio; CI, confidence interval.

Topical ointments for pruritus were used by 21 patients (48.8%) during the control phase and 6 patients (13.6%) during the intervention phase. Topical ointment use was lower during the intervention phase (*χ*^2^ = 12.590, *P* < 0.001), with an RR of 0.28 (95% CI: 0.12–0.62), an RD of −35.20% (95% CI: −53.26% to −17.14%), and an NNT of 2.84 (Table 2).

### 3.4 Exploratory comparison of VAS scores and ointment-use duration among patients who developed incident pruritus during follow-up

Because the assigned massage protocol was maintained after pruritus onset in the intervention group, the following post-onset comparisons may have been influenced by continued intervention exposure and should therefore be interpreted as exploratory.

Among patients who developed pruritus during the observation period, 14 patients in the intervention group and 28 patients in the control group were included in the conditional analysis. The intervention group had a significantly lower VAS score than the control group [3.50 (3.00, 4.00) vs. 5.00 (5.00, 6.00); Z = −3.64, *P* < 0.001]. The duration of ointment use was also significantly shorter in the intervention group [0.00 (0.00, 4.75) vs. 7.50 (3.00, 9.00) days; Z = −3.08, *P* < 0.001] (Table 4).

**Table 4:** VAS scores and duration of ointment use among patients who developed pruritus during the observation period.

Item	Intervention Group (*n* = 14)	Control Group (*n* = 28)	Statistic Z	*P*-value
VAS score (points)	3.50 (3.00,4.00)	5.00 (5.00,6.00)	−3.64	<0.001
Ointment use duration (days)	0.00 (0.00,4.75)	7.5 (3.00,9.00)	−3.08	<0.001

Values are presented as median (P25, P75). Only patients who developed pruritus during the observation period were included in this analysis (intervention group, *n* = 14; control group, *n* = 28). Between-group comparisons were performed using the Mann–Whitney *U*-test, with two-sided exact *P* values reported.

### 3.5 Incidence of adverse reactions in the intervention group

During the study period, two of the 44 participants in the intervention group developed mild local skin reactions (4.5%). Patient 1 was a 56-year-old woman who developed a 3 × 4 cm area of localized erythema accompanied by mild worsening itching and burning sensations over both anterior shins on day 5 after starting the intervention. Massage was discontinued, and cold compresses and moisturizing care were provided; the reaction resolved within 3 days. Patient 2 was a 61-year-old woman who developed a 2 × 3 cm area of localized erythema with mild stinging over both extensor forearms on day 3. Massage was discontinued and cold compresses were applied; the reaction resolved within 2 days. Neither patient underwent component-specific testing or controlled rechallenge; therefore, the responsible component could not be identified. Following evaluation by the research team and attending medical personnel, both events were considered possibly related to hypersensitivity or irritation related to exposure to the essential-oil preparation. This assessment was based on the temporal relationship between application of the preparation and symptom onset, together with the recognized contact-sensitizing potential of tea tree oil. The intervention was temporarily discontinued, and the symptoms resolved following observation and appropriate symptomatic management. However, because component-specific patch testing and controlled rechallenge were not performed, tea tree oil allergy could not be definitively confirmed.

## 4 Discussion

### 4.1 Interpretation of the observed between-phase differences

In this single-center, prospective, non-concurrent before-and-after study of maintenance hemodialysis patients without pruritus at baseline, the intervention phase showed lower observed rates of incident CKD-aP and topical ointment use than the control phase. However, intervention status was completely aligned with calendar period. The differences may therefore reflect seasonal environmental changes, the composite intervention, or both, and the independent contribution of essential oil massage cannot be determined.

The magnitude of the observed association warrants particular caution. Pruritus occurred in 65.1% of patients during the control phase and 31.8% during the intervention phase, corresponding to an unadjusted absolute risk difference of 33.3 percentage points, a relative risk of 0.49, and an apparent number needed to treat of approximately 3. This association is larger than that reported for some pharmacological treatments evaluated in randomized trials. In the multicenter, double-blind, placebo-controlled KALM-1 trial, 49.1% of patients treated with difelikefalin and 27.9% receiving placebo achieved a reduction of at least 3 points in the Worst Itching Intensity Numerical Rating Scale at 12 weeks, an absolute between-group difference of approximately 21 percentage points (7). Although that trial addressed treatment of moderate-to-severe pruritus rather than prevention, the comparison underscores the need for cautious interpretation. Seasonal confounding, participants' expectations, additional attention and coaching, subjective reporting, and unblinded outcome ascertainment may all have contributed to the observed association. The risk difference should therefore not be interpreted as evidence that essential oil massage alone produced a biological effect of this magnitude. A multivariable logistic regression sensitivity analysis adjusting for history of pruritus, serum phosphorus, and primary disease yielded a consistently lower odds of incident pruritus in the intervention phase (adjusted OR = 0.217, 95% CI: 0.084–0.559; *P* = 0.002). The association between study phase and incident pruritus therefore persisted after adjustment for these measured baseline covariates. However, the category-specific estimate for diabetic nephropathy was imprecise (adjusted OR = 4.042, 95% CI: 1.031–15.849), and the overall Wald test for primary disease was not statistically significant (*P* = 0.248); this contrast may therefore reflect small-sample instability and should not be interpreted as an independent association between diabetic nephropathy and incident pruritus. Notably, URR was marginally higher in the control phase than in the intervention phase (67.73% vs. 66.17%; SMD = 0.181), an imbalance that, if greater dialysis adequacy independently reduces the risk of CKD-aP, would be expected to bias against rather than favor the intervention phase. However, residual confounding from unmeasured factors and seasonal effects cannot be excluded.

Among participants who developed pruritus, the intervention phase also showed lower VAS scores and shorter ointment-use duration. These conditional analyses involved small, unequal subgroups and were not the basis of the sample-size calculation. Moreover, the 1.5-point difference in median VAS score was below the commonly cited minimal clinically important difference of approximately 2 points. These findings are exploratory, and their clinical relevance remains uncertain.

### 4.2 Possible explanations for the observed between-phase difference

CKD-aP is not solely a localized skin disorder, but reflects interacting systemic, inflammatory, neural, opioid-receptor, and skin-barrier processes (8). Recent literature has emphasized the importance of xerosis and impaired barrier function in CKD-aP and the potential value of barrier-restoring skin care (9). If the composite intervention influenced the observed outcomes, the most plausible local explanations would be support of skin hydration through the oil-based vehicle and modulation of itch perception through gentle tactile stimulation. Regular lubrication might reduce dryness and interrupt the cycle of itching, scratching, and further barrier damage (10), whereas sustained tactile input might alter the sensory processing of itch (11), These explanations concern the composite intervention and do not establish a component-specific effect.

Previous aromatherapy and massage studies in hemodialysis populations have generally enrolled patients with existing pruritus and evaluated symptom relief rather than prevention. A systematic review of eight studies involving 461 hemodialysis patients reported an overall beneficial effect on pruritus, but the evidence was limited by small samples, methodological weaknesses, and substantial heterogeneity (12). A randomized trial also suggested that massage and plant-oil preparations may both contribute to symptom relief (13). These treatment studies provide only indirect context and should not be interpreted as evidence that essential oil massage prevents new-onset CKD-aP.

Mechanistically, CKD-aP involves uremic toxins, immune-inflammatory abnormalities, opioid-receptor imbalance, altered peripheral and central sensory processing, and skin-barrier impairment (14). Regular application of an oil-based preparation could support local moisturization and barrier integrity before the onset of symptoms. In a cross-sectional study of 162 maintenance hemodialysis patients, Chang and Liao found that pruritus was associated with stratum corneum hydration and transepidermal water loss (15). However, the present study did not measure skin hydration, transepidermal water loss, inflammatory markers over time, or other mediators; it therefore cannot demonstrate that barrier improvement accounted for the between-phase difference.

Gentle rhythmic tactile stimulation might influence itch perception through peripheral, central, and neuroimmune sensory pathways (16). However, sensory modulation was not directly measured and cannot explain whether the intervention prevented the onset of CKD-aP. Because massage was maintained after pruritus onset, this potential mechanism is relevant only to the exploratory post-onset symptom-severity findings and should not be interpreted as evidence of preventive efficacy. The preparation also combined three essential oils with sweet almond oil, so any contribution from an individual component cannot be inferred. Because the study lacked comparator groups receiving massage with a neutral carrier oil or equivalent attention without massage, the contributions of massage, carrier oil, essential oils, and additional contact could not be separated; the specific contribution of the essential oils was therefore unestimated.

### 4.3 Implementation and safety considerations

The intervention was delivered by trained family caregivers, with nurses providing standardized instruction, supervision, and follow-up. This caregiver-delivered, nurse-supported model required no complex equipment and was incorporated into the participants' existing dialysis care. Reviews and consensus documents emphasize regular emollient use, dialysis optimization, standardized symptom screening, and sustained patient education as components of CKD-aP management (2, 17). Practical reviews likewise recommend incorporating symptom assessment and multidisciplinary support into routine care rather than relying only on patients to report symptoms spontaneously (18). The present study shows that the protocol could be initiated and monitored under supervised study conditions, but it did not formally measure staff time, caregiver burden, acceptability, cost, or implementation fidelity.

Previous studies in hemodialysis populations have reported that caregiver- or nurse-supported aromatherapy massage can be delivered within structured care settings (19). Other evidence suggests that aromatherapy interventions may be integrated into dialysis-related symptom management (20). However, these studies addressed different symptoms and intervention formats and do not establish feasibility or effectiveness for preventing CKD-aP. The ability to provide training and follow-up in the present study should therefore be interpreted as preliminary implementation experience rather than evidence for routine clinical adoption.

Two participants developed mild localized reactions that resolved after discontinuation and supportive care. These observations describe short-term events only. Adverse reactions were actively assessed only during the intervention phase, so no concurrent background rate was available and comparative safety cannot be established. The 24-hour tolerance procedure assessed immediate irritation rather than delayed sensitization, and the short follow-up did not address reactions related to repeated or oxidized essential-oil exposure. Longer-term safety would require standardized exposure records, systematic assessment in all comparison groups, and component-specific evaluation when clinically indicated.

### 4.4 Limitations

The one-time enrollment procedure may have introduced selection bias because the two study phases did not necessarily represent the same underlying population of prevalent hemodialysis patients. Although patients enrolled during the control phase were not eligible for re-enrollment during the intervention phase, other patients who remained on the dialysis roster but had not previously participated could enter the study when they first met the eligibility criteria during the intervention period. Consequently, the two phases were drawn from structurally different source pools: the intervention-phase pool excluded patients previously enrolled during the control phase, including those who remained free of pruritus, whereas patients who had not previously been enrolled and who became symptom-free and otherwise eligible by the intervention period could enter the intervention phase. This asymmetry may have altered the distribution of underlying susceptibility to pruritus between phases and constitutes an additional source of residual selection bias. The reasons for first becoming eligible or consenting to participate may have been related to unmeasured characteristics, including symptom vulnerability, family support, health stability, or willingness to undertake the intervention. These factors were not systematically measured and therefore could not be fully controlled. Consequently, comparable measured baseline characteristics do not exclude residual selection bias, and the observed between-phase difference should be interpreted as an association between the intervention period and outcomes rather than as a causal effect of essential oil massage.

The study also has several other limitations. First, its single-center, non-randomized, non-concurrent before-and-after design is highly vulnerable to temporal trends, seasonal variation, residual confounding, and Hawthorne effects. Although URR was available as a measure of dialysis adequacy, Kt/V was not collected, which limited a more comprehensive assessment of dialysis adequacy and the evaluation of potential dialysis-related confounding. Because no formal *a priori* sample size calculation was performed and the final sample size was determined by enrolment during the predefined study periods, the modest sample size may have limited statistical precision, particularly for secondary and exploratory analyses. Objective indoor temperature, humidity, and individual heating exposure were not systematically recorded, and no concurrent or historical seasonal control was available. Because intervention exposure was completely aligned with calendar period, the intervention effect cannot be separated from the seasonal effect.

Second, incident pruritus was determined using a study-specific binary patient report rather than a validated CKD-aP instrument. Although pruritus occurrence was assessed weekly, the exact week of first onset was not retained in an analyzable form; therefore, the temporal pattern of incident pruritus could not be evaluated. Participants and outcome assessors were aware of phase assignment, so expectation effects, subjective reporting, and unblinded ascertainment may have exaggerated the observed association. Future studies should use validated instruments such as the WI-NRS or 5-D Itch Scale and blinded independent outcome assessment.

Third, the intervention phase included demonstration, tolerance testing, written instructions, closer monitoring, and retraining that were not provided during the control phase. This attention and coaching imbalance was a differential co-intervention, not a mechanism specific to essential oils. Without neutral-oil massage and attention-matched comparator groups, the effects of massage, carrier oil, essential oils, and additional contact cannot be distinguished.

Fourth, the intervention depended on designated family caregivers and required an ongoing time commitment, which may limit the applicability of this intervention to patients living alone or without consistent family support. Because neither the phase-specific distribution of pre-enrollment exclusions nor caregiver-related non-enrollment was recorded separately, the extent of differential selection between the two phases, including selection associated with caregiver availability, could not be quantified. Adherence was assessed mainly by self-report and nurse follow-up; session-level completion was not systematically recorded, so actual exposure, adherence rate, and dose-response relationships could not be evaluated.

Fifth, the 4-week follow-up was insufficient to evaluate sustained effects or cumulative sensitization. Tea tree and lavender oils may become sensitizing after oxidation, but component-specific testing, formal chemical stability assessment, and controlled rechallenge were not performed. Adverse-event monitoring was limited to the intervention phase, precluding comparative safety conclusions.

Finally, health-related quality of life, sleep quality, and mood were not assessed; consequently, the study cannot determine whether the observed between-phase differences translated into broader patient-centered benefits. Topical ointment prescribing was not protocol-standardized, and closer contact during the intervention phase may have influenced prescribing or reporting. Because the assigned massage protocol was maintained after pruritus onset and the application site was adjusted to the predominant symptomatic region, subsequent VAS scores and ointment-use duration may have been influenced by post-onset intervention exposure. These outcomes should therefore be interpreted only as exploratory measures of symptom experience after pruritus onset and not as indicators of preventive efficacy. Larger multicenter randomized trials with concurrent, attention-matched controls, longer follow-up, validated outcome instruments, and comprehensive patient-centered assessments are required.

## 5 Conclusion

Among maintenance hemodialysis patients without pruritus at baseline, the intervention phase showed a lower observed incidence of new-onset CKD-aP and less use of topical symptomatic ointments than the control phase. Analyses of VAS scores and ointment-use duration among patients who developed pruritus during follow-up were exploratory and should not be interpreted as evidence of a treatment effect. However, as the control and intervention phases were conducted during different calendar periods (January–June vs. July–December), seasonal factors—particularly winter-associated dry air and indoor heating, which may worsen pruritus—cannot be excluded as contributing to the observed differences. Because intervention exposure was completely aligned with calendar period, the present study cannot distinguish an intervention effect from a seasonal effect. Accordingly, the observed difference should not be interpreted as evidence that essential oil massage prevents CKD-aP.

## Data Availability

The raw data supporting the conclusions of this article will be made available by the authors, without undue reservation.
